# The unique second wave phenomenon in contrast enhanced ultrasound imaging with nanobubbles

**DOI:** 10.1038/s41598-022-17756-1

**Published:** 2022-08-10

**Authors:** Chuan Chen, Reshani Perera, Michael C. Kolios, Hessel Wijkstra, Agata A. Exner, Massimo Mischi, Simona Turco

**Affiliations:** 1 Eindhoven University of Technology, Eindhoven, The Netherlands; 2 Case Western Reserve University, Cleveland, OH USA; 3 Ryerson University, Toronto, Canada

**Keywords:** Biophysics, Cancer, Biomarkers, Molecular medicine, Nanoscience and technology, Biomedical engineering

## Abstract

Investigation of nanobubble (NB) pharmacokinetics in contrast-enhanced ultrasound (CEUS) at the pixel level shows a unique phenomenon where the first pass of the contrast agent bolus is accompanied by a second wave. This effect has not been previously observed in CEUS with microbubbles. The objective of this study was to investigate this second-wave phenomenon and its potential clinical applications. Seven mice with a total of fourteen subcutaneously-implanted tumors were included in the experiments. After injecting a bolus of NBs, the NB-CEUS images were acquired to record the time-intensity curves (TICs) at each pixel. These TICs are fitted to a pharmacokinetic model which we designed to describe the observed second-wave phenomenon. The estimated model parameters are presented as parametric maps to visualize the characteristics of tumor lesions. Histological analysis was also conducted in one mouse to compare the molecular features of tumor tissue with the obtained parametric maps. The second-wave phenomenon is evidently shown in a series of pixel-based TICs extracted from either tumor or tissues. The value of two model parameters, the ratio of the peak intensities of the second over the first wave, and the decay rate of the wash-out process present large differences between malignant tumor and normal tissue (0.04 < Jessen-Shannon divergence < 0.08). The occurrence of a second wave is a unique phenomenon that we have observed in NB-CEUS imaging of both mouse tumor and tissue. As the characteristics of the second wave are different between tumor and tissue, this phenomenon has the potential to support the diagnosis of cancerous lesions.

## Introduction

Among the available ultrasound (US) imaging modes, contrast-enhanced ultrasound (CEUS) is widely used for the detection and quantification of blood perfusion^1,2^. Following the intravenous administration of ultrasound contrast agents (UCAs) into the bloodstream, CEUS allows for higher-sensitivity detection of slow blood flow in the microvasculature as compared to conventional color or power Doppler modes^3^. To this end, dedicated contrast-specific imaging sequences have been adopted that drive the MBs close to their resonance frequency and leverage their highly nonlinear response as compared to tissue to enhance the contrast-to-tissue ratio^4^. Currently, the most common UCAs consist of stabilized microbubbles (MBs), where inert gas with low diffusivity is encapsulated in a monolayer phospholipid shell^5^. With a typical diameter of 1–10 µm^5^, MBs are intravascular agents. After injecting MBs into the bloodstream, time-intensity curves (TIC) can be obtained by measuring the US signal from a local MB concentration as a function of time. The extracted TICs usually exhibit a rapid first pass and relatively weaker recirculation passes of the injected MBs within a time range of 1 min. Hemodynamic parameters of clinical interest can be extracted from dedicated TIC analysis. The clinical adoption of CEUS has supported and enabled a broad range of applications, ranging from cancer localization^6^ to myocardial perfusion^7^ up to selective drug delivery by targeted MBs and therapeutic applications^8^. The use of MBs for cancer localization is mostly based on the assessment of perfusion features in the abnormal tumor microvasculature as a result of angiogenesis^6,9^. Differently, dynamic contrast-enhanced (DCE)-MRI and contrast-enhanced CT mainly assess the permeability of angiogenesis and leaky vessels for cancer detection^10^. In fact, the large diameter of MBs, comparable with red blood cells, prevents MB extravasation into the interstitial space, thus hampering their use for assessment of vascular permeability and quantification of biomarkers in the extravascular space^11^.

Nanobubbles (NBs) are regarded as new-generation UCAs whose diameter is more than ten times smaller than conventional MBs^12,13^. Despite being off-resonance, several studies have demonstrated that NBs can produce high nonlinear contrast enhancement using the standard frequency employed in the clinical US^14,15^. NBs are also proven to be able to extravasate from the vasculature into the interstitial space during their extended life span in the blood circulation^12,14,16^. The prolonged retention effect owning to NB extravasation can be used to detect an increase in microvasculature permeability, hence serving as an additional biomarker for the detection of cancer angiogenesis^17^. Furthermore, similar to MBs, the NB shell can be decorated to target specific membrane proteins, such as prostate-specific membrane antigen (PSMA), so as to bind to receptors that are overexpressed in cancer cells^18,19^. Given the diameter of NBs, mostly in the range of 100–300 nm, it is reasonable to expect that the pharmacokinetics of NB differs from that of larger MBs as well as from that of smaller molecular contrast agents commonly used in MRI and CT, such as gadolinium and iodine. In existing studies, the NB pharmacokinetics was indirectly assessed by examining the TIC extracted from a large ROI^12,14^. From a more general perspective, the analysis of the NB pharmacokinetics can be considered within the category of nanoparticles (NPs) which have similar diameters and reasonably similar properties as NBs^20^. Their small diameter paves avenues for several diagnostic and therapeutic applications, such as cancer localization and drug delivery^21,22^.

In previous studies, the pharmacokinetic analyses of NPs (or NBs) were mostly qualitative, assessed in temporal intervals of hours or days^23^, and probed at the organ level^24^. The observed TIC from a large ROI is mostly made up of a first rapidly increasing phase and a second slow decaying phase. By utilizing NBs-based CEUS (NB-CEUS), we can achieve both a high frame rate and spatial resolution to facilitate the observation of NB pharmacokinetics with greater detail. Different from the conventional approach based on TIC assessment from a large ROI^25^, analysis of NB-CEUS TICs at the pixel level can bring more insights into the in-vivo pharmacokinetics of NBs.

In this study, we report, to the best of our knowledge for the first time, the occurrence of a second-wave phenomenon unique to NB pharmacokinetics. The first pass of the UCA bolus is, in fact, accompanied by the appearance of a second wave within a time range of about 15 min. This phenomenon has not been observed with conventional MBs. We hypothesized that this second-wave phenomenon is a characteristic of NB pharmacokinetics within the tissue. In this study, we focused on the analysis of the second-wave phenomenon observed in NB-CEUS, proposing a model for its description and possible utilization in clinical applications. Our proposed model incorporates the second-wave phenomenon to fit pixel-based TICs extracted from NB CEUS loops.

## Materials and methods

### NB preparation

In our experiment, we utilized a recently developed NB-UCA whose shell structure is engineered to achieve a higher stability and prolonged half-life during CEUS imaging^26^. For producing this type of NBs, a mixture of glycerol (Gly, Acros Organics) and phosphate buffer saline (PBS) (0.8 mL, Gibco, pH 7.4) was added to a lipid solution (10 mg mL^ − 1^). The mixed solution was then sonicated for 10 min at room temperature and transferred (per 1 mL) to a 3 mL headspace vial. The air inside the vial was manually replaced by octafluoropropane (C3F8, Electronic Fluorocarbons, LLC, PA) gas three times. The phospholipid solution was then activated by mechanical agitation of the vial with a VialMix shaker (Bristol-Myers Squibb Medical Imaging Inc., N. Billerica, MA) for 45 s. The NBs were then isolated from the mixture of bubble population by centrifugation at 50 g force for 5 min. Readers can refer to^26^ for more details about the preparation of NBs. In this study, the diameter distribution and concentration of NBs were characterized with a Resonant Mass Measurement (RMM, Archimedes, Malvern Instruments)^27^. The diameter of the obtained NBs was measured to be 281 ± 2 nm. The average concentration of NBs was estimated to be 4.07 + /− 0.25 × 10^10^ NBs per mL.

### Animal model

A total of seven 4–6-weeks old athymic nude mice were employed for acquiring CEUS images. In each mouse, a dual-tumor model, with one PC3flu tumor on the left side and one PC3pip (overexpressing prostate-specific membrane antigen, PSMA) tumor on the right side, was initiated by subcutaneously injecting 1 × 10^6^ PC3flu and PC3pip cells in 100 μL matrigel. This dual-tumor model was used in our previous study for investigating the prolonged retention effect due to the specific binding of PSMA-targeted NBs^28^. Apart from the PSMA expression, we hypothesized that there is no apparent difference in the vasculature and morphology between the two types of tumor. Retrovirally-transformed PC3pip cells and transfection-control PC3flu cells were originally obtained from Dr. Michel Sadelain (Memorial-Sloan Kettering Cancer Center, New York, NY) and were obtained for this study from the laboratory of Dr. James Basilion at CWRU. The mice were observed every other day until one tumor’s diameter reached 8–10 mm. Before acquiring the CEUS loops, the mice were anesthetized by inhalation of 3% isoflurane with 1 L/min oxygen. In this study, mice were handled according to a protocol approved by the Institutional Animal Care and Use Committee (IACUC) at Case Western Reserve University (CWRU) in accordance with all applicable protocols and guidelines. All methods and experiments were performed in accordance with the relevant guidelines and regulations.

### CEUS imaging

**Figure 1:**
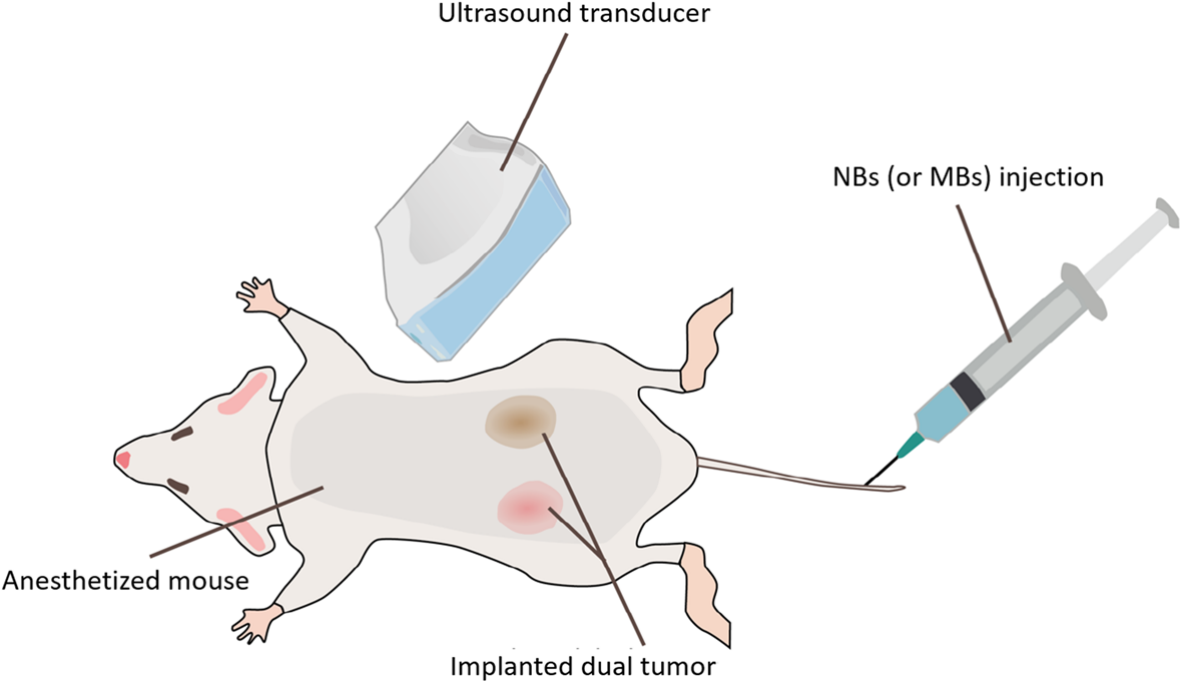
Schematic illustration of the experimental setup. The male athymic nude mice were anesthetized before acquiring CEUS images. A dual tumor was implanted in each mouse subcutaneously in each limb. The ultrasound transducer was fixated at a position at which both tumors were in the field of view. CEUS imaging was performed by administering a 200-μL bolus of NBs (or MBs for reference) via the tail vein.

For acquiring CEUS imaging, 200-μL NBs prepared as above were administrated over 30–40 s via the tail vein using a 26 G catheter. A PLT-1204BT probe (central frequency, 12 MHz; MI, 0.1; dynamic range, 65 dB; gain, 70 dB; imaging frame rate, 0.2 frames/s) connected to a clinical ultrasound machine (AplioXG SSA-790A, Toshiba Medical Imaging Systems) was fixed prior to CEUS imaging to reduce motion artefacts. CEUS acquisitions were performed to image the left PC3flu tumor and the right PC3pip tumor ROI in the same field of view. The CEUS image has a pixel size of $112\mathrm{ \mu m}$. The spatial resolution was estimated to be about $250\mathrm{ \mu m }\times 150\mathrm{ \mu m}$ (lateral $\times$ axial) by measuring the average intensity profiles of several imaging points. Due to storage limitations in the US scanner and to avoid excessive destruction of the contrast agent, the first 5 min the CEUS loops were recorded at 1 Hz, after which the sampling rate was switched 0.2 Hz. After about 30 min, high-intensity flashes were employed to destroy the remaining NBs. We assumed that the majority NBs are cleared out since the residual contrast enhancement dropped to baseline level after the high-intensity flashes^12^. The experimental setup of the CEUS acquisition is illustrated in Fig. 1. In one mouse, Lumason MBs (sulfur hexafluoride lipid-type A microspheres, Bracco Diagnostics Inc.,) were also administrated for acquiring conventional CEUS 30 min after the NB-CEUS (1 h after first injection, the contrast reached the baseline level), as a reference to be compared with the NB-CEUS. Our previous studies have confirmed that the prepared NBs and Lumason MBs can produce comparable maximum in-vivo enhancement^26,28^.

### Data processing and TIC extraction

After acquiring the NB-CEUS videos, the evolution of the dB-scaled contrast enhancement over time was recorded to obtain a TIC at each pixel. To improve the signal-to-noise ratio, TICs were spatially filtered by convoluting the dB-scaled images with a 2D Gaussian kernel (Full-Width-at-Half-Maximum, FWHM, 300 µm). The initial spatial resolution of CEUS images was estimated to be about 250 μm × 150 μm (lateral × axial) by measuring the average intensity profiles of several imaging points. By applying the isotropic kernel with FWHM of 300 μm, which is larger than the spatial resolution, the filtered CEUS images present higher signal-to-noise ratio and more uniform resolution. The spatially filtered dB-scaled TICs were then linearized into intensity-scaled TICs by reverting the dynamic-range compression and color mapping implemented in CEUS^29^. A linear relationship between the image intensity and the local concentration of UCAs has been reported in many studies^30^. The TICs were then resampled at 1 Hz frequency and filtered by a low-pass filter with a passband frequency of 0.1 Hz for further analysis.

### TIC modeling

The preprocessed, pixel-based TICs were fitted to a new model that incorporates the second-wave phenomenon. This model is fundamentally based on the assumption of a convective-dispersion process of the NBs, as given by the modified local density random walk (mLDRW) model^6^, with the addition of a retention compartment, already proposed to describe the transport of targeted MBs^31^. We have extended this model by doubling its parameterization of the convective-dispersion process to also describe the second-wave phenomenon. The resulting model is therefore referred to as double-mLDRW model. By the double-mLDRW model, the local NB concentration, $C\left(t\right)$, is considered as the superimposition of two waves, the first pass of the contrast agents and a second wave that occurs at least three minutes after UCA administration. Each wave is composed of one intravascular transport function and one additional retention function. The transport function is described by the mLDRW model as^6^

1 $${C}_{mLDRW}\left(t;{t}_{0}, \kappa ,\mu \right)=AUC\sqrt{\frac{\kappa }{2\pi \left(t-{t}_{0}\right)}}{e}^{-\frac{\kappa {\left(t-{t}_{0}-\mu \right)}^{2}}{2\left(t-{t}_{0}\right)}},$$

where ${t}_{0}$ is the theoretical injection time, $\mu$ represents the mean transmit time, $AUC$ denotes the area under the curve, and $\kappa$ represents a local dispersion-related parameter given by $\kappa ={v}^{2}/D$, with $v$ being the NB intravascular velocity and D the NB intravascular dispersion. The retention function is modeled by the convolution between the intravascular function and an exponential function, representing the extravascular compartment^32,33^. The resulting analytical expression of the double-mLDRW model is

2 $$\begin{gathered} C\left( t \right) = \alpha _{1} \left[ {C_{{mLDRW}} \left( {t;t_{0} ,\kappa _{1} ,\mu _{1} } \right) + \beta _{1} C_{{mLDRW}} \left( {t;t_{0} ,\kappa _{1} ,\mu _{1} } \right)*e^{{ - \lambda t}} } \right] \hfill \\ \quad \quad \quad \quad + \alpha _{2} \left[ {C_{{mLDRW}} \left( {t;t_{0} ,\kappa _{2} ,\mu _{2} } \right) + \beta _{2} C_{{mLDRW}} \left( {t;t_{0} ,\kappa _{2} ,\mu _{2} } \right)*e^{{ - \lambda t}} } \right] \hfill \\ \end{gathered}$$

Here, the function ${C}_{mLDRW}\left(t;{t}_{0}, \kappa ,\mu \right)$ represents the mLDRW model that describes the intravascular transport process; ${\kappa }_{1}$ and ${\mu }_{1}$ represent the dispersion-related parameter and mean transit time, respectively; ${\alpha }_{1}$ and ${\beta }_{1}$ represent intensity scaling ratios of the first wave and the retention function for the first wave, respectively. Likewise, ${\kappa }_{2}$, ${\mu }_{2}$, ${\alpha }_{2}$ and ${\beta }_{2}$ denote the corresponding parameters for the second wave. $\lambda$ represents the decaying rate of retention function. Specifically, we denote the maximum values of the first wave and the second wave as

3 $$\begin{gathered} {\text{m}}_{1} = max\left\{ {\alpha_{1} \left[ {C_{mLDRW} \left( {t;t_{0} ,\kappa_{1} ,\mu_{1} } \right) + \beta_{1} C_{mLDRW} \left( {t;t_{0} ,\kappa_{1} ,\mu_{1} } \right)*e^{ - \lambda t} } \right]} \right\}, \hfill \\ {\text{m}}_{2} = max\left\{ {\alpha_{2} \left[ {C_{mLDRW} \left( {t;t_{0} ,\kappa_{2} ,\mu_{2} } \right) + \beta_{2} C_{mLDRW} \left( {t;t_{0} ,\kappa_{2} ,\mu_{2} } \right)*e^{ - \lambda t} } \right]} \right\}, \hfill \\ \end{gathered}$$

respectively. The time to peak of the first wave and the second wave are represented by ${\tau }_{1}$ and ${\tau }_{2}$, respectively.

It can be noticed that the two waves share the same the same theoretical injection time, ${t}_{0}$, and decaying rate, $\lambda$, for mitigating the risk of overfitting as well as to provide a more realistic representation of the underlying transport-retention process. Physiologically, the theoretical UCA injection time, ${t}_{0}$, is expected to be the same for the two waves; the decaying rate $\lambda$ of the retention function is mainly determined by the vascular permeability, which is also expected to be the same for the two waves.

Curve fitting was performed by a least-square fitting method using the trust-region reflective algorithm, as implemented in the Python SciPy package^34^. The initial values, as well as the upper and lower bounds for each parameter, were approximated based on the measured time to peak (TTP), peak enhancement (PE), area under the curve (AUC), and wash-out rate (WoR). Based on our observations, the range of possible ${\mu }_{1}$ values was bounded between 1 to 2 min, and the range of the possible ${\mu }_{2}$ values was bounded between 3 to 17 min. For the parameter ${t}_{0}$, we implemented an iterative search grid spanning from 45 to 75 s in 1-s steps to search for the smallest squared error. Pixels with a maximum grey level lower than 3 were excluded from the TIC fitting to guarantee adequate levels of signal-to-noise ratio. The goodness of model fitting was evaluated by the coefficient of determination ${R}^{2}$ defined as:

4 $${R}^{2}=1-\frac{{\Vert {C}_{e}\left(t\right)-C\left(t\right)\Vert }^{2}}{{\Vert {C}_{e}\left(t\right)-\overline{{C }_{e}}\Vert }^{2}}$$

where ${C}_{e}(t)$ $C\left(t\right)$, and $\Vert \cdot \Vert$ represent the measured TIC, the double-mLDRW fitted TIC, and the Euclidean norm, respectively.

### Evaluation of the model parameters

The TIC model fits were evaluated by both quantitative and qualitative assessments. After fitting pixel-based TICs with the proposed double-mLDRW model, parametric maps of $\lambda$, ${\mathrm{log}}_{10}\left({m}_{2}/{m}_{1}\right)$, ${\tau }_{2}$, and ${\kappa }_{2}$ were produced and visualized. $\lambda$ represents the decaying rate which is affected by NB extravasation and vasculature structure. ${\mathrm{log}}_{10}\left({m}_{2}/{m}_{1}\right)$ indicates the peak intensity of the second wave relative to the first wave. ${\tau }_{2}$ and ${\kappa }_{2}$ represent the characteristics of the second wave in terms of time to peak and convective-dispersion kinetics. Since the untargeted nanobubbles are used in this study, we consider the pharmacokinetics in the two types of tumor to be equivalent. Hence, we make no distinctions between parameters in the two types of tumor ROIs in the following evaluations. The locations of the two tumors were roughly delineated by two elliptical-shape ROIs based on both B-mode and CEUS images. For each tumor ROI, an elliptical ROI of the same diameter, orientation, and depth as the ROI in the tumor was selected to represent normal tissue. The distance between the tumor and tissue ROIs was set to 75% of the length of the long axis. The hypo-enhanced area (maximum quantized CEUS level <  = 3) in the filtered image was also excluded from the tissue ROI. Considering the available image content within the field of view, the middle area between two tumor ROIs appears to be the most reasonable choice for selecting the two ROIs with diameters that are comparable to those of the tumor ROIs. Moreover, the tissue ROI mainly includes the spinal cord in the upper part and muscle in the lower part. These two organs are both known to have dense vasculature with a typical hierarchical vascular branching structure^35,36^, which is different from the abnormal tumor vasculature^37^. Including these two organs in the tissue ROIs can provide a suitable reference in comparing vasculatures between healthy and cancerous tissues. Because the spatial filtering kernel size and spatial resolution are larger than the pixel size, the parametric maps were spatially down-sampled by two times in each direction for suppressing the sample correlation caused by the large spatial resolution and filtering kernel diameter.

To evaluate the overall parameter difference, we adopted the Jessen-Shannon divergence (JSD)^38^ with Bootstrap test^39^ as a metric for quantifying the difference of the parameter value distributions in tumor and tissue ROIs. To empirically calculate JSD, the probability distributions of the parameter values in tumor and tissue ROIs were estimated from the histogram, consisting of 50 bins ranging from 0.5% quartile to 99.5% quartile of all parameter values (pooling parameter values in both tumor and tissue ROIs). For realizing the Bootstrap test, the pooled pixel-level parameter datasets were randomly resampled (with repetitions) to create bootstrapped datasets with 1/12 the size of the original datasets (since 12 pairs of tumor-tissue ROIs were included in the evaluation). This resampling process was repeatedly performed for 2000 times to generate the bootstrapped datasets for calculating 2000 JSD values. Based on these bootstrapped JSD values, the 95% confidence interval of JSD for differentiating parameters in one tumor ROI and one tissue ROI can be then estimated. Furthermore, because the distribution of pixel-level parameters within a ROI does not usually follow a symmetric parametric distribution, we adopted a non-parametric strategy to evaluate the estimated parameter distributions by comparing the 25% quartile, median value (50% quartile), and 75% quartile of the parameter values in tumor and normal tissue ROIs. Additionally, two JSDs, 25% quartile, median value (50%), and 75% quartile of the parameters in the left and right pairs of tumor-tissue ROIs were respectively calculated for each mouse. We compared the relative difference in cumulative probability distributions between tumors and tissues for both the pooled parameter values and the parameter values for each mouse. As the maximum difference exceeds one quartile (25%, termed a one-quartile difference), the parameter difference was considered significant. The criterion of one-quartile difference was adopted in several previous studies from different fields^40–42^. The qualitative evaluation was performed by visualizing the parametric maps and a number of pixel TICs, together with the model fit, demonstrating the second-wave phenomenon at the pixel level. Moreover, to show that the second-wave phenomenon can be observed not only in the pixel’s range but also in a local area on the CEUS image, we visualized the average TICs from two representative rectangular ROIs of 900 pixels, with one located in the highly enhanced region of the left tumor and one in the lowly enhanced region of the right tumor in the exceptional mouse. The average process can also efficiently improve the signal-to-noise ratio of TIC in the lowly enhanced region, especially for the MB-based CEUS.

### Histological analysis

Histological analysis was conducted in one exceptional mouse to examine the molecular characteristics of the two tumors. This exceptional mouse was also imaged with both NB-CEUS and CEUS with conventional MBs. After the CEUS acquisitions, Phosphate-buffered saline (PBS) perfusion was performed with 50-mL PBS through the left ventricle. After the PBS perfusion, the mice were euthanized by the exposure to CO_2_. The two tumor lesions were then harvested and embedded in the optimal cutting temperature compound (OCT Sakura Finetek USA Inc., Torrance, CA). The tissues were parallelly cut into 9-µm slices close to the acquisition plane. Although the histological section may not represent the exact same acquisition plane, the anatomical structures presented in the histological section are overall consistent with the landmarks observed on CEUS. CD31 staining was then performed to visualize the tumor vessels. Briefly, the tissue slices were washed 3 times with PBS and incubated with a protein blocking solution that contain 0.5% Triton X-100 (Fisher Scientific, Hampton, NH). Tissue slices were incubated in 1:250 diluted CD31 primary antibody (Fisher Scientific, Hampton, NH) for 24 h at 4 °C. After being washed with PBS, the tissue slices were incubated with Alexa 568 tagged secondary antibody (Fisher Scientific, Hampton, NH) for one hour and stained with DAPI (Vecor Laboratories, Burlingame, CA) using standard techniques. Fluorescence images were observed under Leica DM4000B fluorescence microscopy (Leica Microsystem Inc, Buffalo Grove, IL). For comparison with the CEUS images, the fluorescence and histological images were cognitively matched with CEUS images. This alignment between fluorescence and histological images was realized by visually identifying the anatomical landmarks presented on histological slices and CEUS images. The detailed images of corresponding anatomical landmarks are presented in the Supplementary materials. On the CD31 staining image, one core ROI with about > 1 mm distance from the tumor border and the other rim ROI with about < 1 mm distance from the tumor border were delineated for each tumor. The average intensities and standard deviation of CD31 staining in the two ROIs were calculated for comparison.

### Ethical approval

Mice were handled according to a protocol approved by the Institutional Animal Care and Use Committee (IACUC) at Case Western Reserve University (CWRU) in accordance with ARRIVE guidelines.

## Results

**Figure 2:**
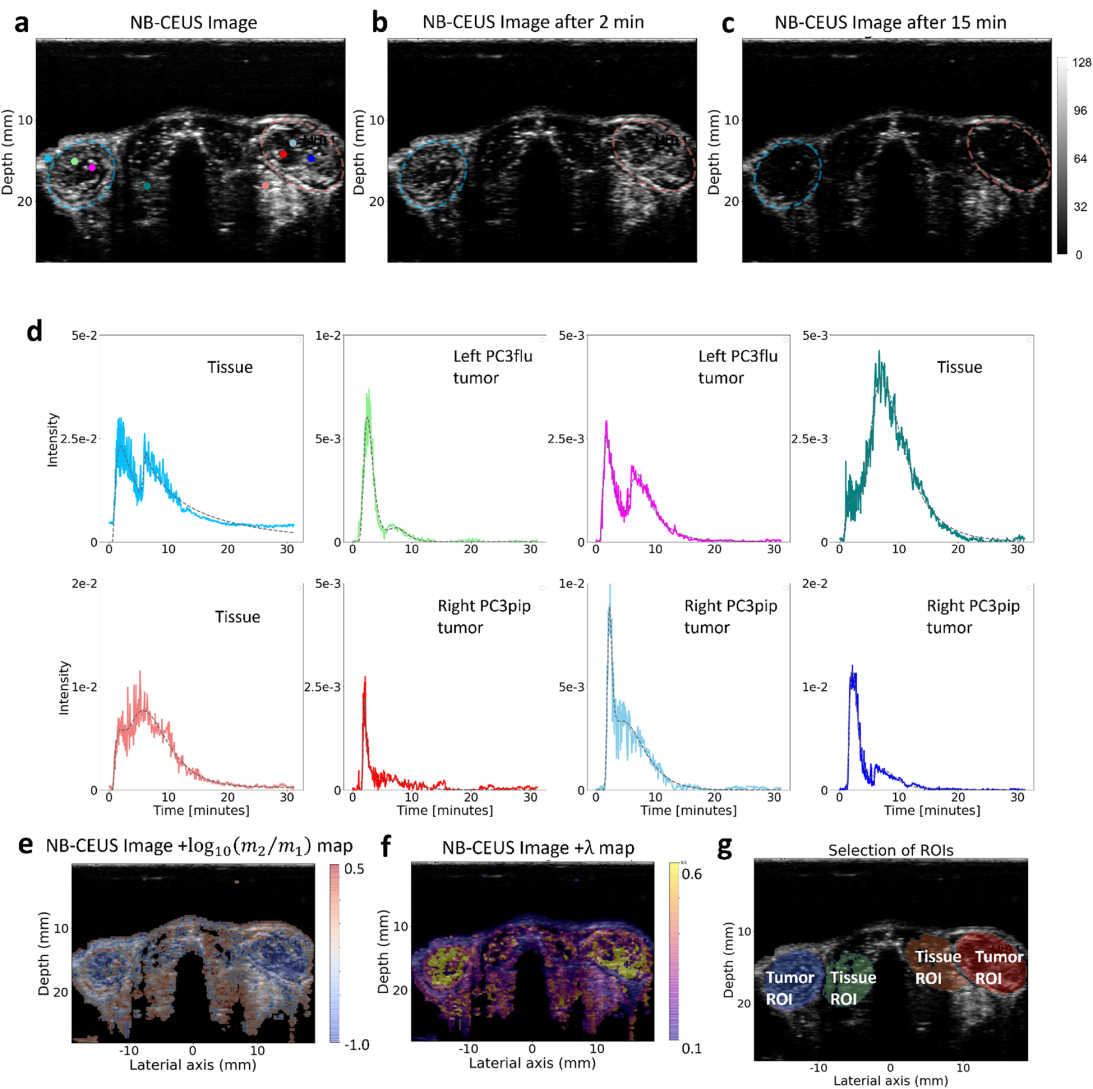
An example of pixel-based TICs and parametric maps in mouse dual-tumor model. (**a**) presents the NB-CEUS image of overall highest enhancement, with the left PC3flu and right PC3pip tumor ROIs delineated in blue and red, respectively. The NB-CEUS images acquired at time 2 and 15 min after the injection are displayed (**b**) and (**c**), respectively. In (**d**), a series of experimental pixel-based TICs (colored solid lines) and corresponding model fit (gray dashed lines) are extracted from different locations which are indicated by the corresponding colored dots in (**a**). The classification of each dot’s location is labelled out. The scales of y-axis varies between subfigures in (**d**). (**e**) and (**f**) present the overlays of parametric maps of ${log}_{10}\left({m}_{2}/{m}_{1}\right)$ and $\lambda$ upon the NB-CEUS projection image, respectively. In (**g**) the ROIs corresponding to tumor and tissue are indicated by colored masks.

**Figure 3:**
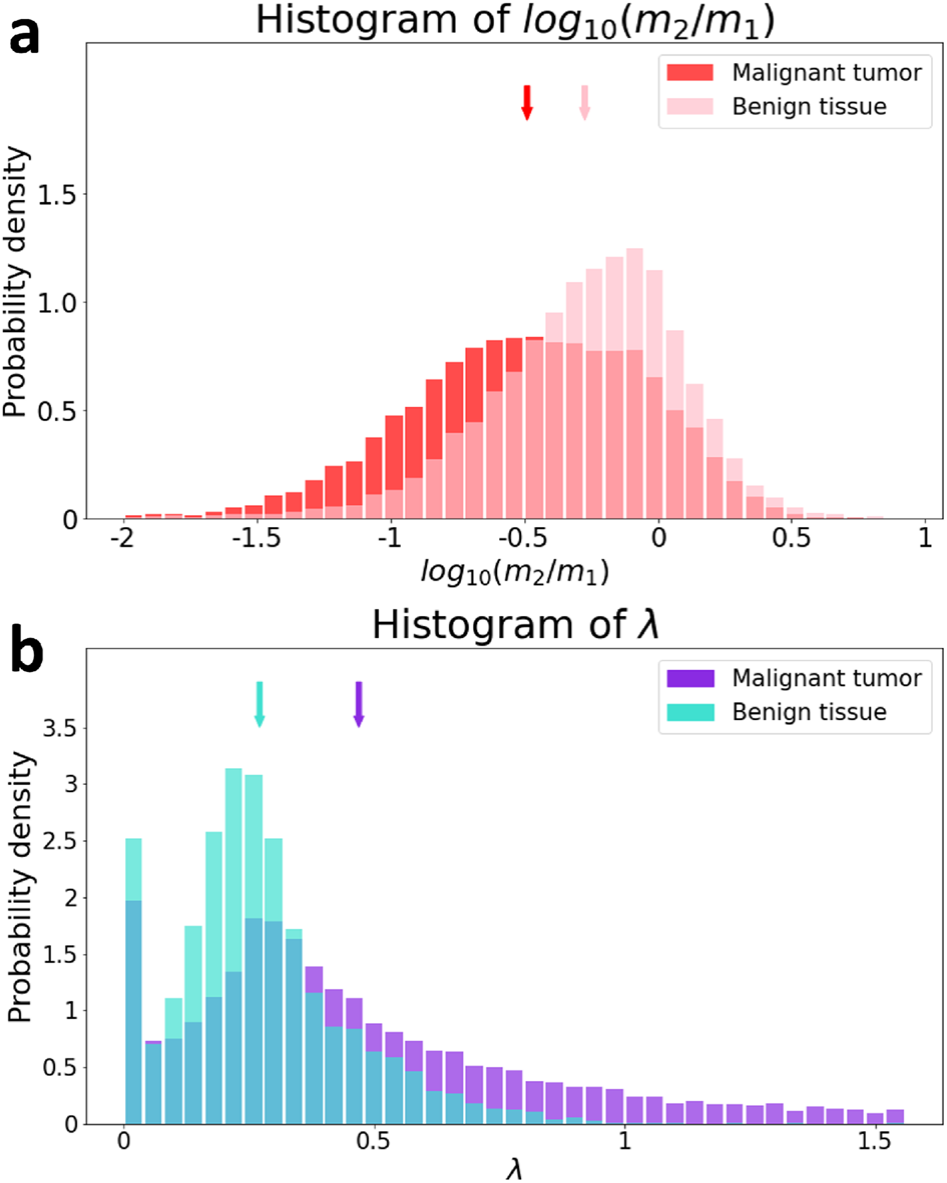
Histogram of parameters ${log}_{10}\left({m}_{2}/{m}_{1}\right)$ and $\lambda$ values between the tumor and tissue. (**a**) displays two histograms of ${log}_{10}\left({m}_{2}/{m}_{1}\right)$ values collected from twelve tumor ROIs (total of ~ 12,900 pixels) and twelve reference tissue ROIs (total of ~ 8000 pixels) in six mice. (**b**) displays the corresponding two histograms for the $\lambda$ values. The mean values of these parameters within the tumor ROIs and tissue ROIs are indicated by arrows of corresponding colors.

The model-fitting results of one dual-tumor mouse case are shown in Fig. 2. The second-wave phenomenon can be observed in the majority of pixel-based TICs. The parametric maps of both ${\mathrm{log}}_{10}\left({m}_{2}/{m}_{1}\right)$ and $\uplambda$ exhibit a clear difference between the tumor lesion and the tissue outside the tumor. Without loss of generality, the parameter values over twelve tumors from six mice (excluding the mouse where MB-CEUS was acquired) are pooled together and presented in Fig. 3. The 95% confidence interval of $\mathrm{JSD}$ for quantifying the differences between the pooled parameter values, and the 25% quartile, median value, and 75% quartile of ${\mathrm{log}}_{10}\left({m}_{2}/{m}_{1}\right)$, $\uplambda$, ${\kappa }_{2}$ and ${\tau }_{2}$ in tumor and normal tissue ROIs are presented in Table 1. Lower ${\mathrm{log}}_{10}\left({m}_{2}/{m}_{1}\right)$ values and higher $\uplambda$ values with one-quartile difference (marked in bold) were obtained in tumor lesions compared to the surrounding tissues. Focusing on the $\uplambda$ values, there were 25.5% high values ($\uplambda >0.6$) found in tumors in comparison with the 3.3% high values ($\uplambda >$ 0.6) presented in tissues. Table 2 lists the 25% quartile, median value, and 75% quartile of ${\mathrm{log}}_{10}\left({m}_{2}/{m}_{1}\right)$ and $\uplambda$ values in tumor and normal tissue ROIs for each mouse. The parameters with one-quartile difference between tumor and tissue are marked in bold. The $\mathrm{JSD}$ for quantifying the parameter difference in tumor and tissue ROIs for each mouse is also listed. A number of TICs collected from tumors and tissues are presented in Fig. 2 to show the second-wave phenomenon at the pixel level. For the total of six mice, 86% of pixel-based TICs were well fitted with the double-mLDRW model with ${R}^{2}>0.9$.

**Table 1:** Jessen–Shannon divergence ($\mathrm{JSD}$) and quartiles for quantifying the differences of parameter values.

	JSD		25% quartile	Median value	75% quartile
${{\varvec{l}}{\varvec{o}}{\varvec{g}}}_{10}\left({{\varvec{m}}}_{2}/{{\varvec{m}}}_{1}\right)$	0.046–0.071	Tumor	−0.779*	**−0.479**	**−0.159**
		Tissue	**−0.472**	**−0.230**	**−0.025**
${\varvec{\lambda}}$	0.069–0.080	Tumor	**0.228**	**0.367**	**0.619**
		Tissue	**0.163**	**0.249**	**0.350**
${{\varvec{\kappa}}}_{2}$	0.022–0.030	Tumor	1.358	1.737	3.409
		Tissue	1.146	1.511	2.354
${{\varvec{\tau}}}_{2}$	0.015–0.020	Tumor	6.022	6.852	8.113
		Tissue	5.807	6.752	8.013

*The bolded description represents that there is a one-quartile difference in parameter values between tumor and tissue.

**Table 2:** Median value, two quartiles, and JSD of parameter values for each mouse.

		${log}_{10}\left({m}_{2}/{m}_{1}\right)$					$\lambda$				
		25% quartile	Median value	75% quartile	JSD left tumor	JSD right tumor	25% quartile	Median value	75% quartile	JSD left tumor	JSD right tumor
Mouse 1	Tumor	−0.532*	**−0.243**	**−0.010**	0.119	0.060	**0.264**	**0.351**	**0.509**	0.076	0.083
	tissue	**−0.212**	**−0.057**	**0.107**			**0.196**	**0.260**	**0.341**		
Mouse 2	Tumor	**−0.845**	**−0.562**	**−0.278**	0.067	0.132	**0.239**	**0.344**	**0.645**	0.126	0.109
	Tissue	**−0.451**	**−0.235**	**−0.044**			**0.167**	**0.233**	**0.306**		
Mouse 3	Tumor	**−0.909**	**−0.612**	**−0.261**	0.133	0.044	**0.303**	**0.457**	**0.730**	0.281	0.108
	Tissue	**−0.602**	**−0.389**	**−0.101**			**0.044**	**0.181**	**0.301**		
Mouse 4	Tumor	**−0.730**	**−0.410**	**−0.150**	0.070	0.247	**0.161**	**0.282**	**0.585**	0.070	0.104
	Tissue	**−0.276**	**−0.111**	**0.042**			**0.135**	**0.252**	**0.397**		
Mouse 5	Tumor	−0.612	−0.367	−0.149	0.046	0.057	**0.205**	**0.256**	**0.342**	0.280	0.127
	Tissue	−0.536	−0.317	−0.095			**0.029**	**0.165**	**0.305**		
Mouse 6	Tumor	**−0.870**	**−0.636**	**−0.399**	0.079	0.074	**0.250**	**0.583**	**0.843**	0.113	0.112
	Tissue	**−0.693**	**−0.465**	**−0.257**			**0.080**	**0.418**	**0.572**		

*The bolded description represents that there is a one−quartile difference in parameter values between tumor and tissue.

**Figure 4:**
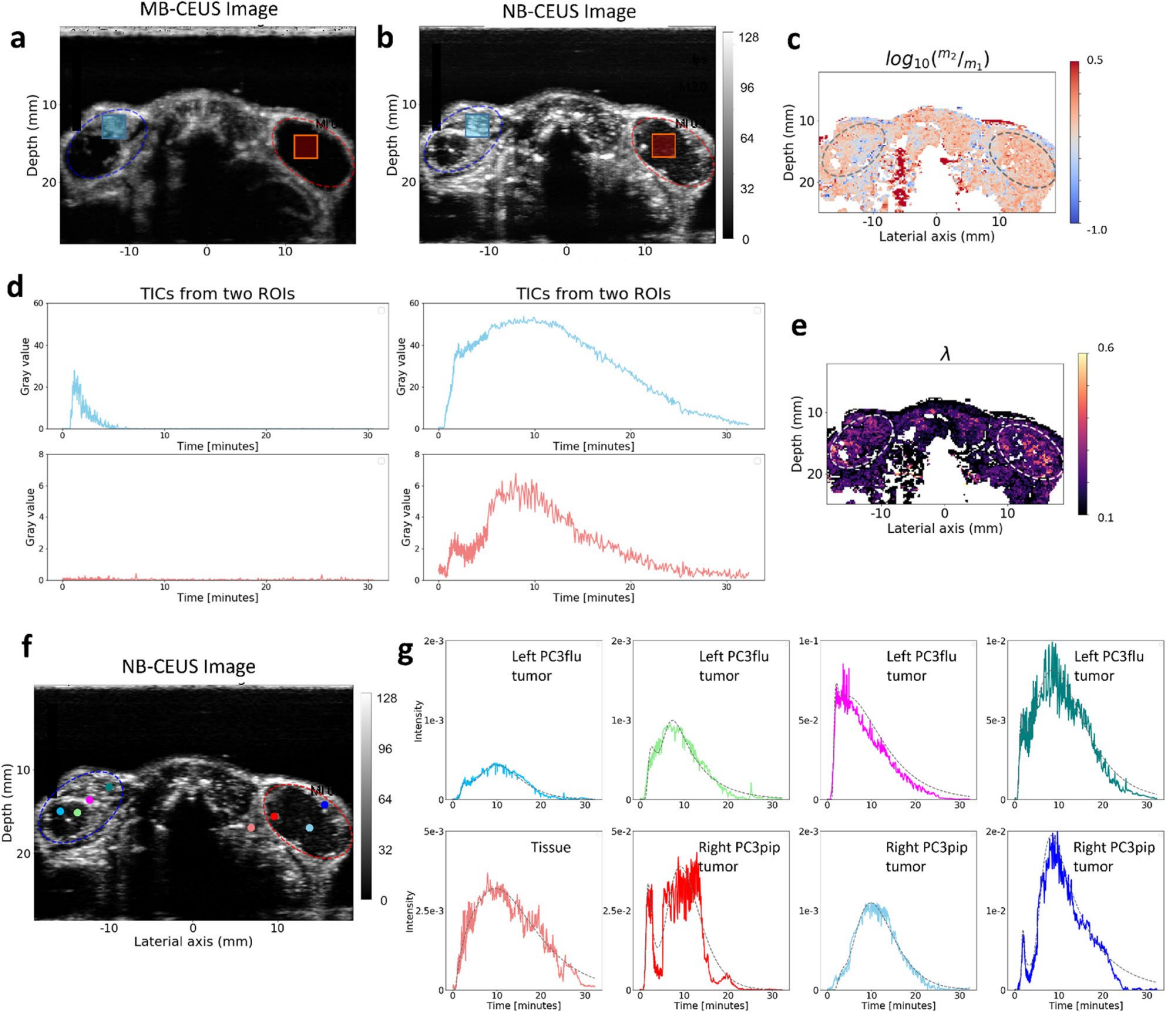
Comparison of parametric maps and TICs obtained on one mouse with MBs and NBs. In the top row, the maximum intensity projection of MB-CEUS (**a**) and NB-CEUS (**b**) videos are presented, with the left PC3pip and right PC3 tumor ROIs delineated in blue and red, respectively. The average TICs extracted from two rectangular ROIs within the tumors are presented in (**d**) with corresponding colors. (**c**) and (**e**) present the parametric maps of ${log}_{10}\left({m}_{2}/{m}_{1}\right)$ and $\lambda$, respectively. In (**g**), a series of experimental pixel-based TICs (colored solid lines) and corresponding model fit (gray dashed lines) are extracted from the NBs-CEUS video, with the pixels’ locations indicated by corresponding colored dots in (**f**). The scales of the y-axis vary between subfigures in (**g**).

Apart from these twelve tumors in six mice, in one mouse both NB-CEUS and MB-CEUS were acquired (see Fig. 4). In comparison with MB-CEUS, NB-CEUS produced a longer period of high contrast enchantment. The region with evident NB perfusion was larger than the region with MB perfusion, especially in the right PC3pip tumor, where NB-CEUS enhancement was high in the peripheral regions and relatively lower in the central regions, while the MBs produced almost no enhancement. In the average TICs collected from two rectangular ROIs of 900 pixels within the two tumors, the presence of a second wave can be clearly recognized in the right ROI (red-colored TIC) and is also visible in the right ROI (blue-colored TIC). For the right PC3pip tumor that presented almost no MB-CEUS enhancement, the estimated ${\mathrm{log}}_{10}\left({m}_{2}/{m}_{1}\right)$ values in the tumor were overall higher than the values in the tissue. This differs from the relatively lower ${\mathrm{log}}_{10}\left({m}_{2}/{m}_{1}\right)$ values observed in the tumor of mice 1–6. As shown in Fig. 4f, the second wave appears to be stronger for the cyan-colored TIC that is extracted from the right tumor. The second wave can be clearly distinguished from the first wave on the red-colored TIC and blue-colored TIC, both extracted from the rim of the right tumor.

**Figure 5:**
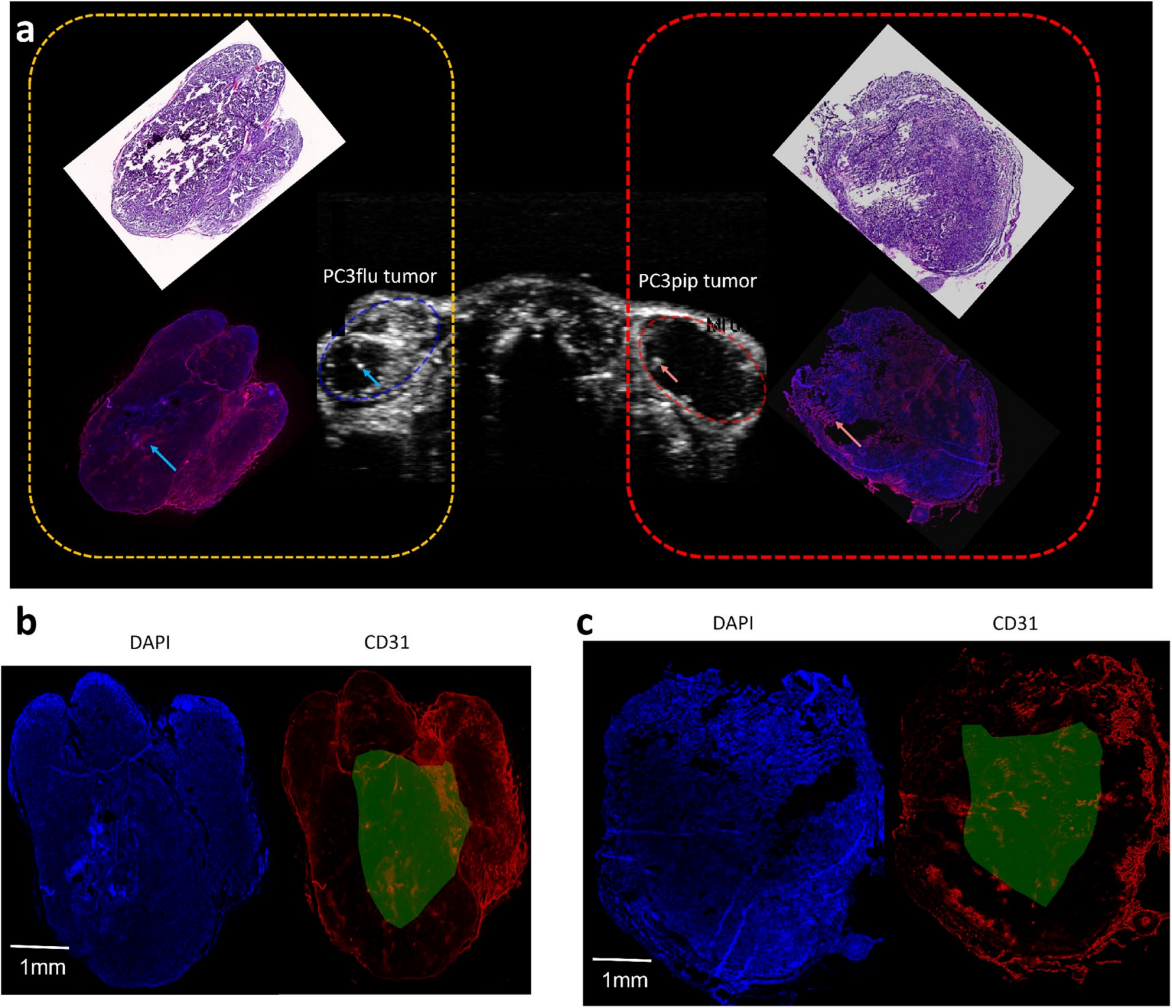
Histology and fluorescence imaging analyses of the dual-tumor model in one mouse. In (**a**), the rotated histology images and fluorescence images that overlay aligned DAPI and CD31 staining images are presented by the sides of corresponding left PC3pip and right PC3flu tumors, Two pairs of colored arrows indicate the correlation between the location of high CD31 staining and the region of high NB-CEUS enhancement. The orientations of histology images and fluorescence images are based on the shape of tumors. The separate DAPI and CD31 fluorescence images of the left PC3pip and right PC3flu tumors are presented in (**b**) and (**c**), respectively. The manually delineated core ROIs for calculating the average fluorescence intensities were indicated by green masks.

To investigate the biological characteristics and especially the vessel distribution in the two tumors, histological analysis of one mouse case is presented in Fig. 5. After aligning the CEUS images with the histological and fluorescence images, we can observe a general agreement between the location of high CD31 staining and the region of high NB-CEUS enhancement in both tumors (see the arrows with corresponding colors to indicate the landmarks). The CD31 staining intensities of the left PC3flu tumor rim and core ROIs were $5.04\pm 4.88$ and $3.56\pm 3.84$ (mean $\pm$ standard deviation), respectively. The CD31 staining intensities of the right PC3pip tumor rim and core ROIs were $3.71\pm 6.02$ and $1.38\pm 3.12$ (mean value $\pm$ standard deviation), respectively. After aligning the CEUS images with the histological and fluorescence images, we can observe a general agreement between the location of high CD31 staining and the region of high NB-CEUS enhancement in both tumors (see the arrows with corresponding colors to indicate the landmarks).

## Discussion

As an alternative imaging modality to MRI and CT, CEUS is a promising diagnostic tool for enhancing specific organ structures with several advantages, such as low cost, flexibility, wide availability, high spatial and temporal resolution. The development of NBs as a new-generation UCA can be generally regarded as a special category of NP-based pharmaceuticals and CAs. Partly due to the limitations of existing imaging modalities, previous analyses of the pharmacokinetics of NPs were mostly performed at the organ level, without considering local characteristics of the NP kinetics. In this work we demonstrated the possibility of assessing the TICs in NB-CEUS at the pixel level (~ 100 μm), resulting in the generation of parametric maps. Considering the image spatial resolution and spatial filtering kernel, the resolution of the parametric maps is approximately 300 μm. In our NB-CEUS experiment in mice, we observed a unique 2nd-wave phenomenon mainly occurring in the time range of 3 to 15 min after contrast injection. The overall peak intensity of the second wave often exceeds the intensity of the first wave. To our best knowledge, this second-wave phenomenon has not been described before in the literature describing NPs and NBs. For conventional small-molecular CA in MRI and CT, the shape of pixel-based TICs is typically composed of a the rapidly-rising phase due to CA vascular transport, and a slowly-decaying monotonous phase or plateau due to the extravasation of the contrast agent. In MB-CEUS, a second rise of contrast enhancement due to blood recirculation mainly occurs within 1 min after the first UCA pass (first wave). However, the intensity of this recirculation wave is generally about 5 times lower than the intensity of the first wave^43^. Given the high intensity (usually exceeding the peak intensity of first wave) and the relatively late occurrence of the second wave (typically reaching the peak between 3 and 15 min after the contrast appearance in the CEUS images), it is unlikely to ascribe the formation of the second wave to intravascular recirculation in the same manner as for MB-CEUS. Instead, the formation of the second wave is more likely to be caused by the in-vivo kinetics of NBs within the internal vasculature and organ structures.

Based on existing pharmacokinetic modeling^32,34^, we proposed the double-mLDRW model, by which the variation in NB concentration at the pixel level is described by the superimposition of two waves, both following the mLDRW model extended with an extravascular compartment. By fitting the pixel-based TICs with the proposed double-mLDRW model, we can estimate several parameters and visualize them as parametric maps to analyze the different NB pharmacokinetics in tumors and tissue. Our results showed that the estimated ${\mathrm{log}}_{10}\left({m}_{2}/{m}_{1}\right)$ value is significantly lower in tumors than in tissue. This suggests that a relatively weaker second-wave phenomenon occurs in tumors in comparison to the surrounding tissue. The parametric maps of $\lambda$ showed the decaying rate of the NB concentration to be overall more prominent in the tumor than in the surrounding tissue. These differences can be probably ascribed to the abnormal tumor vasculature caused by angiogenesis. Different from the normal vasculature that exhibits a regular branching with a typical arteriole-capillary-venule hierarchy, tumor vasculature usually lacks this hierarchical structure and presents irregular and chaotic branching. To support tumor growth, tumor angiogenesis yields a vascular network with large functional blood vessels, higher microvascular density, tortuous morphology, excessive branching, abnormal shunts, and increased permeability^44,45^. The larger functional blood vessels and abnormal shunts could guide more NBs to flow through large vessels and circumvent the slower perfusion in the capillary bed. The high microvascular density, blood shunting, and excessive branching can probably facilitate the washout process of NBs. A faster washout process in tumors in comparison to tissues was also observed in studying the in-vivo kinetics of MBs ^6^. The combination of these abnormal features can possibly contribute to the observed relatively weaker second wave and a faster wash-out process in tumors. The other factors, e.g. NBs extravasation from the leaky vessels, likely also play roles in altering the characteristics of the second-wave phenomenon.

Regarding the right PC3pip tumor in the exceptional case with almost no MB perfusion and low NB perfusion, we hypothesize that the core region of this tumor core presented low vessel density and mainly small capillaries, which are evidenced by the low CD31 staining intensities in the histological slice. Due to the low vessel density and small capillaries, it is difficult for MBs to perfuse in the tumor core region efficiently. Probably due to the smaller diameter, NBs can still efficiently perfuse the tumor rim and penetrate the tumor core. The low vessel density and small capillaries can be due to the inadequate vasculature with increased inter-vessel distances and the development of tumor hypoxia^43^. It is worth noting that the evidence of CD31 staining in supporting this hypothesis is limited in two aspects: firstly, the alignment of slice and CEUS imaging plane was not perfect; secondly, the high CD31 fluorescence mainly indicates the presence of large vessels, while the small capillaries are difficult to be clearly identified. These could explain the discrepancy between the NB-CEUS enhancement and CD31 intensities in the left PC3flu tumor.

As shown in the parametric maps of ${\mathrm{log}}_{10}\left({m}_{2}/{m}_{1}\right)$, the regions of the relatively more intensive first wave were mostly correlated with the regions of high contrast enhancement; the regions of relatively higher-intensity second wave mostly overlap with the regions of low contrast enhancement. Based on common knowledge of MB-CEUS, high contrast enhancement usually occurs in large vessels, while low contrast enhancement indicates the absence of large vessels. Hence, we can hypothesize that the second wave mainly represents NB perfusion of the capillary bed and the interstitial spaces; instead, the first wave mainly represents the NB transport in large vessels. As one imaging pixel might contain both large vessels, small capillaries, and interstitial spaces, the TIC of one imaging pixel can be interpreted as the overlay of the fast transport of NBs in large vessels and the slower perfusion in the small capillary bed or interstitial spaces. In the presence of blood shunts in the tumor, the fast transport and slower perfusion of NBs will be gathered in the veins resulting in two well-separated waves. This is one interpretation that can explain the clear separation of two waves in the TICs from the hyper-enhancing rim regions of the right PC3pip tumor in Fig. 3. We hypothesize that the second-wave phenomenon is a combination of the fast NB transport in large vessels and the slow NB perfusion of the capillaries and interstitial spaces, probably also involving the role of blood shunts. In the future, dedicated pharmacokinetic modeling will be developed to provide a more accurate description of the NB pharmacokinetics in large vessels, capillary bed, and interstitial spaces to verify our hypothesis.

Two parameters, e.g., the relative intensities of two waves ${\mathrm{log}}_{10}\left({m}_{2}/{m}_{1}\right)$ and the decaying rate $\lambda$, were significantly different between tumors and tissues. Although not presenting significant differences as ${\mathrm{log}}_{10}\left({m}_{2}/{m}_{1}\right)$ and $\lambda$, the values of other parameters, such as the skewness ${\kappa }_{2}$ and time delay ${\tau }_{2}$ of the second wave, are also worthwhile to be further investigated by improving the quantitative fitting model and physiology analyses. It is also noted that the result in this study is still preliminary and limited to seven mice. The validity of the developed double-mLDRW model needs to be further evaluated and optimized. Furthermore, this study only included the histological analysis of one mouse as a reference. In future studies, more histological data should be acquired to provide additional evidence of the correlation between the characteristics of the second-wave phenomenon and the underlying (immuno)histopathological tissue and vascular characteristics. With regard to the newly observed second-wave phenomenon, there are still several aspects that need further exploration. Primarily, the mechanism underlying the second-wave generation needs to be further researched via both theoretical modeling and experimental validation. Secondly, the roles of several possible factors, e.g., the NB extravasation, distribution of large vessels, the microstructure of the capillary, and NB diameter, in affecting the second-wave phenomenon requires thorough investigation via pharmacokinetic modeling and controlled experiments. Thirdly, in this study, the statistics were separately performed for individual parameters, without considering the possible correlation between parameters. The correlation between parameters and the underlying relevant mechanisms is worth further investigation. Furthermore, it is of value to consider the characteristics of the second-wave phenomenon presented in various organ structures, including different types of tumors in the kidneys, liver, and spleen. Lastly, it remains unclear if the second-wave phenomenon observed in NB-CEUS also applies to the other types of NPs. Briefly, the second-wave phenomenon suggests that the characteristics and potential applications of NBs are expanded compared to those of current MBs.

To conclude, in this study, we presented preliminary results to reveal the unique second-wave phenomenon observed in NB-CEUS. By pharmacokinetic modeling that incorporates the second wave, a number of parametric maps were obtained to analyse the different pharmacokinetics of NBs in tumors and tissues. We obtained significantly different parameter values between tumors and tissues, suggesting the potential application of NBs in cancer diagnostics. The applications, mechanism, and detailed characteristics of the second-wave phenomenon are topics of further investigation.

## Supplementary Information

Supplementary Information.

## Data Availability

The data that support the findings of this study are available from the corresponding author, Simona Turco, upon reasonable request.
