# Supplementary material for: The unique second wave phenomenon in contrast enhanced ultrasound imaging with nanobubbles
Source: Sci Rep. 2022 Aug 10;12:13619. doi: 10.1038/s41598-022-17756-1 (PMC9365822; doi:10.1038/s41598-022-17756-1)
Supplement: Supplementary file 1 — Supplementary Information. [file 41598_2022_17756_MOESM1_ESM.docx]

**Supplementary material**

The data that support the findings of this study are available from the corresponding author, Simona Turco, upon reasonable request.


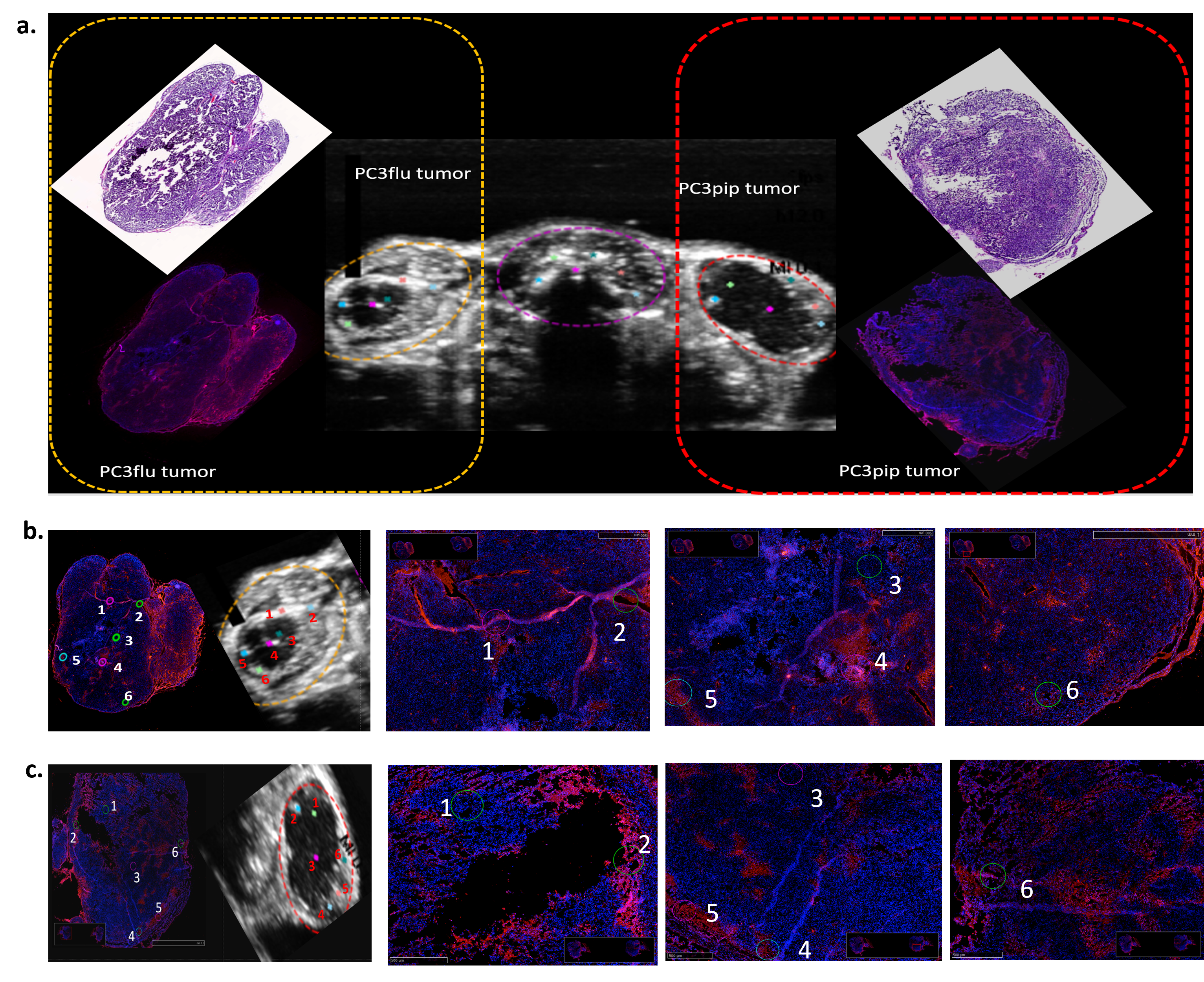


**Figure S1:** Cognitive matching of the histology and fluorescence imaging of the dual-tumor model in one mouse. In (**a**), the rotated histology images and fluorescence images that overlay aligned DAPI and CD31 staining images are presented by the sides of corresponding left PC3pip and right PC3flu tumors. The corresponding six landmarks used for aligning the histology images and fluorescence images of the left PC3pip and right PC3flu tumors are presented in (**b**) and (**c**), respectively.
